# Interior automobile seats: A syntactical and perceptual study

**DOI:** 10.1016/j.heliyon.2024.e29024

**Published:** 2024-04-02

**Authors:** Yingjie Huang, Danhua Zhao

**Affiliations:** a College of Communication and Art Design, University of Shanghai for Science and Technology, Shanghai, 200093, PR China; b School of Design, Hunan University, Changsha, Hunan, 410082, PR China

**Keywords:** Layout of seats of automobile, Fluidity and privacy of interior space, Space syntax, Space perception

## Abstract

This study investigated the seat layout of automobile interiors and its impact on the fluidity and privacy of interior space using spatial perception and space syntax research methods. The interior of an automobile is a typical “miniature” passenger space. First, to explore the perception characteristics of interior space fluidity and privacy across different seat configurations, we conducted a perception experiment on the interior space of seven automobile models with various seat layouts. The depth, connection, global integration degree, and standardized integration degree values were obtained using spatial syntax to perform topological calculations on the experimental automobile models. We conducted a correlation analysis in conjunction with the results of the perception experiment and the spatial syntax analysis. The calculation results of spatial syntax analysis are consistent with the experimental results of perception of automobile interior space layout on the fluidity and privacy. The different layout of automobile seats can affect people's perception on the fluidity and privacy of automobile interior space. At the same time, spatial syntax can provide an effective design analysis tool for the fluidity and privacy of automobile interior space.

## 1 Introduction

Automotive products are unique in that they are closely related to social concepts. Starting with the essential concept of the automotive system Urry [1], argued that automobile travel has become a “quasi-private” global mode of mobility. This mode of transportation refers to the movement of people and objects through the space of automobiles. This idea posits that automotive products can provide people with free and unrestricted private travel space [2], which indicates that both spatiality and privacy are important social attributes of automobiles. Meanwhile, in terms of flexible seat configurations and multipurpose storage, automobile interior designs are evolving toward a living space [3]. However, according to George [3], the interior space of an automobile is an artificial space endowed with social attributes and communication. Therefore, communication and privacy are both important social attributes of automotive interior space [4]. Meanwhile, the interior of an automobile is a typical “miniature” passenger space. Etisson [5] noted that a small space means that people can see all the positions of the space from a location, a typical example of which is a tabletop or room-sized space.

From the perspective of spatial perception, the spatial layout of automobile interiors is an important part of automobile interior design that directly affects people's perceptions of the automobile interior space [6]. Automobile seats are the key point that affects the layout of the interior space of an automobile and are the part of the interior space most closely related to people [7]. Piro et al. [8] argued that the layout of automobile seats and the rotation of the backrest affect the riding comfort, level of communication among passengers, and their feelings. Piro et al. established that, in addition to the physical state of the seat itself, the seat layout and its relative position not only affects seat comfort but also involves the communication state between people in the interior space of the automobile. In design practice, perceptual evaluation is a commonly used evaluation method for automotive interior design [[9], [10], [11]]. Such approach not only uses sensory evaluation indicators such as “elegance” and “high-end,” but also adopts *“fluidity” and “privacy” to describe the perceived spatial quality of the interior space of automobile. Therefore, to explore the perception characteristics of interior space fluidity and privacy across different seat configurations, we first conducted a perception experiment on the interior space of seven automobile models with various seat layouts*.

From the perspective of spatial configuration, the seat layout of an automobile presents a state of “group elements,” that is, the seats act as spatial elements, and the seats form a “group” state according to a certain layout. In the interior space of an automobile, human activities, and behaviors actually revolve around this “group element.” Therefore, seat layout plays a role in shaping the interior space of an automobile and endowing it with function and meaning. Hillier's spatial syntactic theory [12] states that the relationship between these spatial elements and space is essentially an expression of spatial configuration. He proposed that when multiple tangible physical forms, even non-material forms such as language form relationships with each other and form a system, and they will find their “configurations.” Configuration is not limited to architecture; it seems to run through all usage rules and operate in a socialized manner [13]. Similarly, in daily life, people's behavior, communication patterns, and even seating order in the interior space of automobiles reflect a socialized configuration. The physical determining factor of this configuration is obviously determined by the position of the person in the interior space of the automobile, whereas the seat determines the position. Therefore, we derived the depth values, connection values, global integration degree, and standardized integration degree values using spatial syntax to perform topological calculations on the experimental automobile models.

Van Nes argues that as a tool, space syntax cannot analyze place characters, but rather place structures. Furthermore, by analyzing various design scenarios, it can provide interesting insights into the relationships between social, cultural, and economic patterns. Van Nes also posits that the success of space syntax seems to depend on at least two things: a concise definition of space and high degree of falsifiability and validation. The space syntax method's independence of context makes it applicable to all types of built environments, independent of types of societies, political structures, and cultures. Hillier and Hanson consider space syntax to involve understanding “the social and spatial content of social patterning” [14]. Therefore, a correlation analysis coupled with the results of the perception experiment and the spatial syntax analysis was used to prove whether consistency exists.

The study of the fluidity and privacy of automotive interior space concerns the social content of spatial patterns. Contemporary automotive interior designers have fully realized that automotive interior space has a strong social attribute and are well aware that the layout of automobile seats is the key to determining the pattern of the automotive interior space. However, only a few effective theories and tools are currently available. This study investigates the consistency between spatial syntactic analysis tools and perceived evaluations of the fluidity and privacy of automotive interiors.

## 2 Methodology

### 2.1 Perception experiment on the fluidity and privacy of the interior space

We set a perception experiment on the fluidity and privacy of the interior space to investigate the effect of seat layout on the fluidity and privacy of interior space and perception difference between ordinary users and designers using a questionnaire survey method. The research process comprised two steps: experimental design and analysis of the experimental results.

### 2.2 Design of the perception experiment

**Fig. 1:**
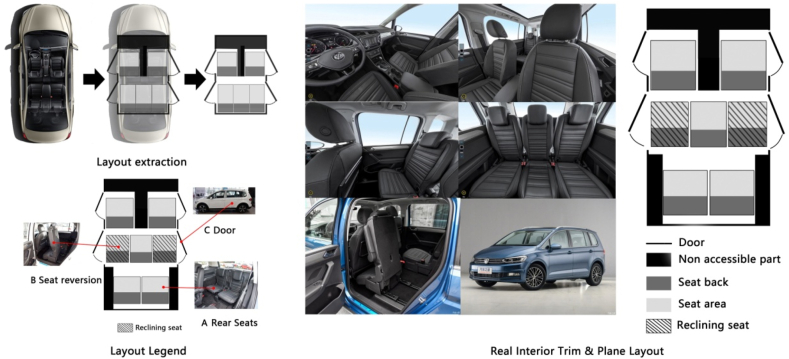
Interior layout extraction and layout legend.

A total of 96 participants (42 university students and 54 from other vocations) participated in a questionnaire survey. The occupation, age, and other basic information of the participants is presented in Table 1. In the experiment, seven models of automobiles with different seats were evaluated: (a) 7-seat Baojun and 7-seat Volkswagen Touran. (b) 5-seat Emgrand GS and 5-seat Hyundai Veloster. (c) 4-seat Audi TT and Mazda RX8. (d) The seventh model was the Mercedes Benz F015 Luxury in Motion (the seat layout of this model automobile can be changed based on the passengers’ requirements. According to the different seat layouts, these were referred to as new, hybrid, and traditional Mercedes Benz). During the experiment, each participant faced panoramic pictures of the interior space of each automobile and the self-painted plan (Fig. 1), including the arrangement of seats, and compared the fluidity and privacy of automobiles with the same seats but with different arrangements. The comparison results were expressed as 1–5 points (best privacy and fluidity = 5 points, = lowest = 1 point). After the validity of the questionnaire data was statistically investigated [15], the scoring results and average values obtained by ordinary participants and those obtained by designers with a design background were counted and are reported in Table 2.

**Table 1:** Basic information of participants in the perception experiment.

	Age	Number	Percentage (%)
Gender	Male	58	60
	Female	38	40
Age distribution	21–25	41	43
	26–35	35	36
	36–40	20	21
Car design background		22	23

**Table 2:** Experiment results of the perception experiment.^a^

Car Model	A	B	C	D	E	F	G	H	I
Fluidity	3.2（3.6）	3.0（3.3）	3.6（3.8）	2.7（3.5）	2.1（2.2）	2.7（3.9）	4.8（4.9）	4.6（4.1）	4.2（4.1）
Privacy	3.0（3.4）	3.3（3.4）	3.0（3.0）	3.2（3.5）	3.8（4.0）	3.8（3.9）	3.2（3.9）	2.3 (3.6)	2.1 (3.7)

^a^(1), automobile models: (A) Baojun; (B) Volkswagen Tuan; (C) Emgrand GS; (D) Hyundai Veloster; (E) Audi TT; (F) Mazda RX8; (G), H0, (I) new, hybrid, traditional Mercedes Benz, respectively. (2) The data in brackets were obtained by common user group.

### 2.3 Statistical analysis of the experimental results

The perception experiment on automobile interior space is the starting point and basis of this study. To test the reliability of the results of the questionnaire survey, the data obtained from the above experiment were investigated using statistical analysis.

#### 2.3.1 Consistency of experimental results between expert and ordinary consumer groups

To examine consistency in the experimental results on the privacy and fluidity of automotive interior space between the expert and general consumer groups, the *t*-test method was used to conduct a significant difference analysis on the data presented in Table 2. According to the method described by Zhang et al. [16], an *F-test* was first used to determine whether a significant difference existed in the evaluation scores of the privacy and fluidity of automotive interiors between the expert and ordinary user groups. The *F*_*c*_ value obtained by calculation was less than *F*_*0.05,8,8*_ (95% confidence level), which indicated that no difference existed in the evaluation of the accuracy of scoring between the groups, that is, a *t-test* could be performed. A *t*-*test* was performed to calculate the scores, and the results are reported in Table 3.

**Table 3:**
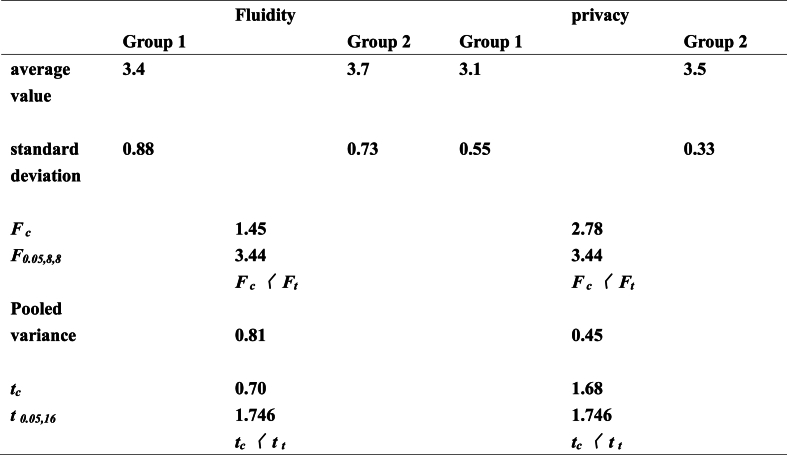
Differences in perception of automotive interior privacy and fluidity between expert and ordinary user groups* * Group 1: expert group; Group 2: ordinary user group. *F*_*c*_: Calculated *F*-value; *F*_*0.05,8,8*_: the value from table, confidence level with 95%. *t*_*c*_*:* Calculated *t*-value; *t*_*0.05,16*_ -value from table, 95% confidence level.

No significant differences were observed between groups. In other words, the expert and general user groups have the same understanding of automobile fluidity and privacy.

**Fig. 2:**
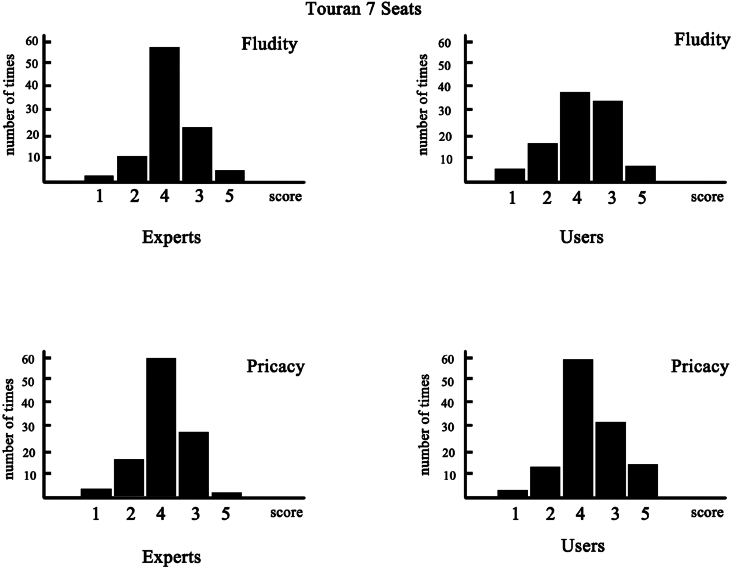
“Space syntax” in the spatial layout of automobile interior.

Fl simplicity, data of other models of automobiles are not shown), is presented in Fig. 2. As shown, the expert and ordinary consumer groups gave relatively centralized scores, which indicates that the statistical distribution and experimental results were reliable. On the other hand, the score concentration degree of the expert group was obviously better than that of the ordinary consumer group, which indicated that the professional background personnel's cognition regarding the automobile characteristics was more comprehensive than that of the ordinary users. According to Table 2, although the scores of the ordinary consumer group were slightly different from those of the professional group, the judgment trend for interior space fluidity was the same as that of the professional group.

#### 2.3.2 Evaluation of experimental results

The following can be derived from the data in Table 2 regarding the automobile interior with same number of seats:

(1) For the Baojun 730 and Volkswagen Touran, although the number of seats was the same, their fluidity scores were 3.2 and 3.0, respectively; the fluidity score of the former was higher than that of the latter. However, the seat arrangement of these two models was different; when passengers want to enter the third row of seats of the Volkswagen Touran, the seat in the second row must be put down, so the fluidity was obviously inferior to that of the Baojun 730. However, the privacy of the Volkswagen Touran was obviously better than that of the Baojun 730 because of the impediment of the second row of seats to the communication of passengers in the back row.
(2) The fluidity score of the Emgrand GS with five seats was higher than that of the Hyundai Veloster; their liquidity scores were 3.6 and 2.7, respectively. The spatial fluidity of the Hyundai Veloster was significantly weaker than that of the Emgrand GS, although the seat layout of the two models is the same. This may be due to the special door setting mode of Hyundai Veloster. Compared with the Emgrand GS, the Hyundai Veloster has fewer doors; the former was designed as one door on the driver’ side and two doors on the passenger's side, resulting in one fewer access channels.
(3) Similar to the situation of 5-seat automobiles, Audi TT with four seats achieved a lower fluidity score than Mazda RX8 (2.1 and 2.7, respectively). The Audi TT with four seats has only two doors, and the Mazda RX8 has the opposite door opening mode; therefore, the fluidity of the Mazda RX8 is obviously better than that of the Audi TT.
(4) The privacy scores of the six automobile models were opposite to their fluidity scores. For the Mercedes Benz F015 Luxury in Motion with 4-seats, the new Mercedes Benz achieved the highest fluidity and privacy scores. Because the seats of the new Mercedes Benz automobile can be turned freely, it showed both good liquidity and privacy.
(5) From Table 2, for the space fluidity values obtained by the expert group, the 7-seat Baojun 730 was better than the 7-seat Volkswagen Touran, Emgrand GS was better than Hyundai Veloster, Audi TT was not as good as Mazda RX8, and the order of their privacy values was the opposite. The order of space fluidity values for the Mercedes Benz F015 Luxury in Motion interior was new seat layout > hybrid seat layout > traditional seat layout. The order of the space fluidity values obtained by the ordinary user group was similar to that obtained by the expert group; however, the overall trend of the results was basically the same for both groups.

### 2.4 Spatial syntax: a graphic method of spatial configuration

From the results of the perception experiment, different seat layouts, orientations, and usage methods have a significant impact on the fluidity and privacy perception of the automobile interior. Further interpreting this phenomenon, the interior of an automobile is an artificial space, and the seats are the most important spatial elements for passengers to interact with. While state of the elements changes affects the overall perception of the space. According to Hillier [12], configuration is not limited to architecture; it seems to run through all usage rules and run in a social manner. When the configuration of one element in the system changes, the configuration attributes of many other elements and the entire system also change. However, the human perception of fluidity and privacy in automobile interior spaces is an instinctive perceptual activity. The question of whether there is a natural and numerical relationship between the state of automobile seats (layout, orientation, etc.) and the perception of interior space is still unclear. In addition, how to use data or certain quantities to help designer complete more intuitive designs in design practice is an important issue in the field [17,18]. To clarify these issues, this study introduced Hillier's spatial syntax to investigate the interior space of various automobile models.

### 2.5 Related terms and explanations of space syntax theory

#### 2.5.1 Adjusted graph (J-graph)

**Fig. 3:**
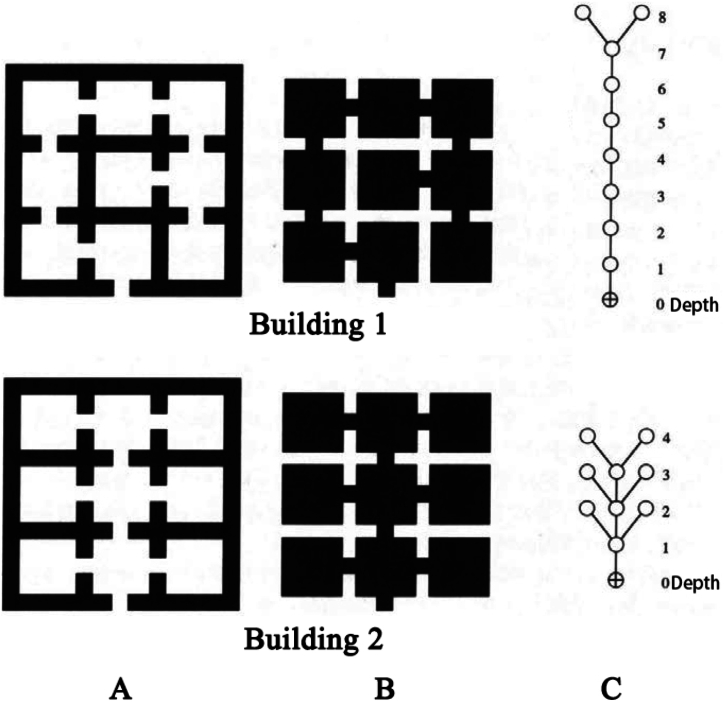
Example of ordinary plane to relation diagram (from Ref. [19]).

The primary research tool for spatial syntax is the J-graph and its corresponding quantitative computations. J-graph, a powerful tool for understanding spatial configuration by applying spatial syntax theory, also known as a relational graph, is a graph of “topological depth” of all spaces starting from a particular point. A classic case to explain the space syntax method is presented in Fig. 3. The first column (Fig. A) on the left shows two building plans, and the black color represents the material entity of the house. The shapes of the two building plans are almost identical; however, the interior partition doors are slightly different. After the first row of plans was inverted to the bottom of the drawing, the two planes of the second row were obtained (Fig. B). Black represents the general spatial layout of the house. In the third column of the corresponding spatial structure, a circle with an internal cross represents the starting point of the diagram, the circle (i.e., node) represents the rectangular space, and the short line indicates the connection between them (Fig. C). As an important way to quantify the configuration J-graph, which is a topological structure diagram, does not emphasize the concepts of distance and shape in Euclidean geometry, but focuses on expressing the structural system composed of the connections between nodes.

### 2.6 Quantitative description of configuration

To quantitatively describe the configuration based on a J-graph, a series of morphological variables based on topological computation were developed in spatial syntax, among which the most basic variables used in this study are as follows [19]:

#### 2.6.1 Connectivity value

Connectivity value is the value assigned according to the number of nodes adjacent to a particular node. In a real-space system, the higher the connection value of a space, the better its spatial permeability.

#### 2.6.2 Depth value

According to the rule that the distance between two adjacent nodes is regarded as a one-step topological space, the depth value represents the shortest distance from one node to another (i.e., the minimum number of steps). The average value of the shortest distance (i.e., the minimum number of steps) from one node to all other nodes in the system is known as the average depth value (*MD*) of the node. It is easy to calculate the average depth value based on J-graph, and the formula can be expressed as $MD=\frac{\sum depth\times number\;of\;nodes\;on\;the\;depth}{total\;number\;of\;nodes\;-\;1}$. For example, in the third column of the first row in Fig. 3, the average depth value of the entrance space is $MD=\frac{1\times 1+2\times 1+3\times 1+4\times 1+5\times 1+6\times 1+7\times 1+8\times 2}{9-1}=5.5$. The total depth of the system is the sum of the average depth of each node. It is obvious that the depth value represents the topological accessibility of the nodes (i.e., the fluidity of the automobile interior space used herein, or the convenience of nodes in the space system). Note that the depth value mainly expresses the number of spatial transformations and not the actual distance.

#### 2.6.3 Integration value (*RA*)

Integration value is related to the number of nodes in the system, calculated using the formula *RA* $=\frac{2\;\left(MD-1\right)\;}{n-2}$, where *n* is the total number of nodes.

#### 2.6.4 Standardized integration degree

Standardized integration degree (*RRA*) can be obtained by taking the reciprocal of *RA*; *RRA* is positively related to the actual meaning and is used to further compare space systems with different sizes. The formula for *RRA* can be expressed as $RRA=\frac{RA}{DN}$, where *DN* is a coefficient $DN=2\;\frac{nlog2\left(\;\frac{n+2}{3-1}+1\right)}{\left(n-1\right)\left(n-2\right)}$. The degree of integration indicates the degree of closeness between nodes and all nodes in the whole system.

According to the above definitions of connection value, depth value, integration degree, and standardized integration degree, for an automobile interior space, the larger the connection value of a certain point, the better the openness of that point, and the less the privacy. The depth value indicates a direct relationship with the fluidity of the automobile interior space; the larger the *MD* value, the more time space conversion occurs and larger *MD* is opposite to the fluidity of people in the interior space. In other words, the communication between passengers is more convenient in the automobile interior space with the reduction of privacy.

**Fig. 4:**
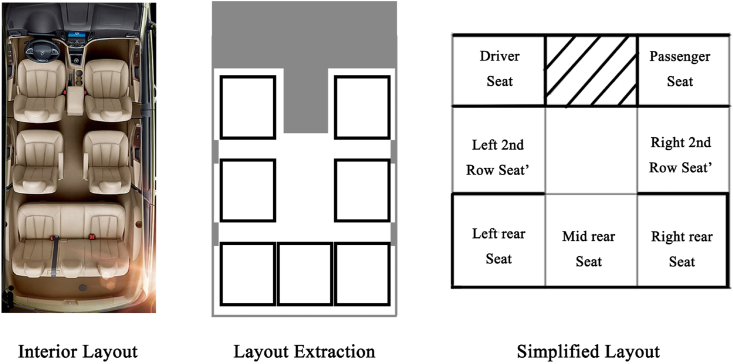
Interior top plan of Baojun 730.

The automobile interior is an enclosed space and the problem regarding this can be discussed from the perspective of “configuration.” In this study, the interior space of an automobile was treated as a plan similar to architecture, and the space was studied using the space syntax research method. In the architecture field, the relationship of interior space can be clarified by that among rooms because it is composed of the relationship between each room, and the activities of humans are mainly around each room. Compared with architecture, an automobile interior can be considered as a moving “room of a building,” and the basic activity space of people in the interior space of automobile is around the seat position and the interior space can be regarded as the configuration. Therefore, the position of the seat can be regarded as the local space of the automobile interior space, that is “room,” and the area around the seat can be regarded as the independent space of the automobile interior space. To clarify the basic relationship of the plane of the automobile interior, a spatial layout extraction graph can be obtained according to the spatial syntax method using the top-view plane of the Baojun 730 interior as an example (Fig. 4). In the “layout extraction” section of Fig. 4, the area of seat is marked with a white box.

Although the passenger space of the automobile interior is different from that of a building that is completely enclosed (rooms of buildings have walls, doors, etc.), each seat space is a semi-closed space, and barriers exist for different passengers; for example, the vision of the passengers in the front and in the back row(s) is different because of the existence of the seat back.

Similar to the effect of various partition walls on architectural space, some functional components in the automobile interior space will also affect the fluidity of the interior space. For example, when a passenger wants to go to the driver's seat from the passenger side, the console and gear lever between the driver's and passenger's seat will make this process very inconvenient. In this case, such a feature is collectively referred to as an obstruction. In the plane layout presented in Fig. 4, the obstacles formed by the control console and gear lever area are marked with a diagonal area, which can clarify the various relationships of the interior space of the automobile.

For the different automobile models, their spatial configurations were different owing to the different interior spaces and different layouts of such space. In essence, the functional requirements of the automobile interior led to the different styling. The changes in the styling of the automobile exterior in different models led to obvious differences in appearance. The spatial layout of the automobile interior not only affects the styling, but also reflects that of the automobile exterior. To a certain extent, the form of “automobile exterior” is the characterization of extensiveness of automobile interior.

### 2.7 Calculation and analysis of space syntax of automobile interior space configuration

The relationship between human activity and spatial modes can be expressed mathematically. Different configuration spaces exhibit different topological depths and the different distribution of spaces. Regarding the relationship between man and space, the space discussed and treated with space syntax in Hillier's book “Space is the Machine” differs from the space that can be measured by mathematical methods of Euclidean geometry, but a relationship can be represented by topological relations. Here, the space syntax relationship is not the actual distance between objects in space, but accessibility and relevance [[20], [21], [22], [23]]. If the automobile interior space is regarded only as a place to complete the functions of automobile products, the attributes of the interior do not to fully express the social functions of the automobile.

The privacy of the individual, communication among passengers in different seats, and fluidity of the passengers in the interior space in the automobile are important characteristics of automobile interiors. The study of space syntax of architecture mainly focuses on the activities of people in the interior space of a building. In the automobile interior with a smaller space compared to the building space, passengers often need to complete tasks such as getting in and out of the vehicle, opening the trunk, temporary parking, or exchanging information between the internal and external space. Therefore, studying the automobile interior space taking the automobile external and interior as a whole is necessary.

In the interior space, where the action behavior of passengers is around the seats, the seat position can be regarded as the basic unit of the interior space, which can be compared to the “room” in architectural space. To investigate the privacy and fluidity of the interior spaces of different automobile brands and models using plane space based on space syntax, each seat in the automobile interior was represented by a hollow square, and the connection mode of the square was the mutual relationship between two seats. Considering the different barriers between the seats in the interior of different automobiles, especially when a passenger sitting in the rear seat wants to go to the driver's seat or passenger seat, it can only be completed after getting out of the automobile. In other words, when the spatial characteristics of an automobile interior are evaluated, the interior and external environment of the automobile must be considered simultaneously. Therefore, when the topological depth of the interior trim was calculated, we determined that one 90° angle turn by the passengers after getting out of the vehicle was regarded as one topological step. For example, when the passengers transfer from the right seat in the second row to the passenger seat, two 90° changes of direction are required after exiting the automobile to reach the front copilot seat (in China, automobile is left rudder), that is, two topological steps are required to complete the transfer from the right seat in the second row to the front passenger seat. In the block diagram (third row in Fig. 4), the topological distance of turning outside the automobile body is represented by a dotted box to distinguish it from the actual seat block; if an obstacle was present between two seats that cannot communicate, this was represented by thick black line. In the case where the front seats must be lowered to reach the rear seats, when the topological depth was calculated, the seat was lowered once was considered as a step of traveling topology. Here, the seat was represented by adding an apostrophe on the upper right of the seat symbol in the block diagram.

**Fig. 5:**
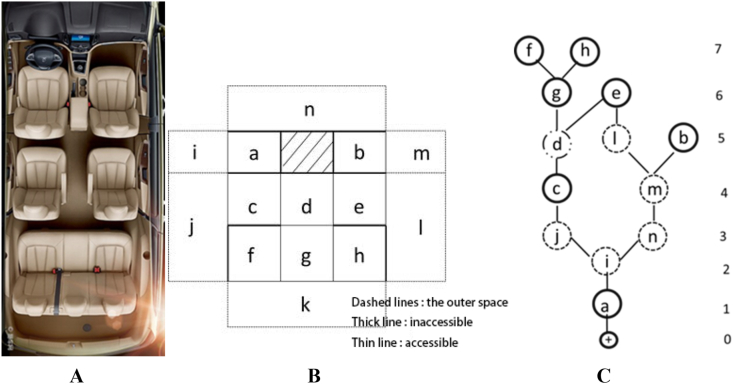
Interior space (a), block diagram (b), and its spatial configuration J-diagram (c): 7- seat Baojun 730.

The J-diagrams of the different models of automobile were examined using the space syntax method. The interior space block diagram and its spatial configuration of the 7-seat Baojun 730 are presented in Fig. 5; the first column (Fig. 5A) shows the interior of 7-seat Baojun 730; and the second column (Fig. 5B) is the block diagram of seat and moving space. Following [19], the third column (Fig. 5C) of Fig. 5 represents the J-diagram with a circle (node) starting from the driver's seat (point a), and the connection between them is represented by a short line. Starting from different positions of the interior seats, different J-diagrams of the same spatial layout can be drawn.

The space syntax method described by Zhang and Wang (2004) was used to calculate the standardized integration degree of the same models of automobile as those used in the perception experiment. The depth value (*MD*), total standardized integration degree (total *RRA*), and other parameters of the different automobile interior spaces obtained by taking the driver's seat, passenger seat, and other positions as starting points are listed in Table 4. Note that the Mercedes Benz F015 Luxury in Motion has passed the road test, its driving mode includes traditional and hybrid modes in which the interior layout can be freely switched, and new mode where four seats can be turned freely; the data of three models are also listed in Table 3. This study revealed the significance of the seat configuration in the design of automobile interior.

**Table 4:** Depth values (MD) of various Automobiles and their standardized integration values (RRA).

Car model	Driver Seat (RRA/MD)	Benz Special Space (RRA/MD)	Passenger Seat (RRA/MD)	Left seat of second row (RRA/MD)	Middle seat of second row (RRA/MD)	Right seat of second row (RRA/MD)	Left seat of back row (RRA/MD)	Middle seat of back row (RRA/MD)	Right seat of back row (RRA/MD)	Total RRA	Total Depth
**A**	0.5870/4.8333		0.5870/4.8333	0.8615/3.7272	0.9941/3.3636^a^	0.8615/3.7272	0.6154/4.8182	1.1236/3.0909	0.6154/4.8182	6.2455	29.8483
**B**	0.7183/4.4167		0.7183/4.4167	0.8338/3.8182	0.8416/3.9167	0.8338/3.8182	0.7014/4.5000		0.7014/4.5000	5.3486	29.3865
**C**	0.6240/4.2222		0.4620/4.2222				0.8048/3.9000	0.7476/3.6250	0.8048/3.9000	3.0652	19.8694
**D**	0.4757/5.1250		0.6830/4.1250				0.4617/5.2500	0.4906/5.0000	0.6244/3.7500	2.7354	23.2500
**E**	0.3809/4.5000		0.3109/4.5000				0.4579/4.7500		0.4579/4.7500	1.6776	18.5000
**F**	0.6528/3.7850		0.6528/3.7850				0.7136/3.7500		0.7136/3.7500	2.7297	15.2500
**G**	0.5146/3.2000	0.6777/2.6000	0.5146/3.2000				0.5146/3.2000		0.5146/3.2000	2.7433	18.0000
**H**	0.4518/3.4000	0.6777/2.6000	0.4518/3.4000				0.4518/3.4000		0.4518/3.4000	2.4849	18.8000
**I**	0.5146/3.2000	0.6777/2.6000	0.5146/3.2000				0.4518/3.4000		0.4518/3.4000	2.6141	15.8000

*A, Baojun 730; B, Touan; C, Emgrand GS; D, Hyundai Veloster; E, Audi TT; F, Mazda RX8; G, New mode of Mercedes Benz; H, traditional model of Mercedes Benz; I, hybrid model of Mercedes Benz.

a Benz Special Space.

## 3 Discussion and analysis

### 3.1 Comparison of the results of perception experiments and computed values of spatial syntax

**Fig. 6:**
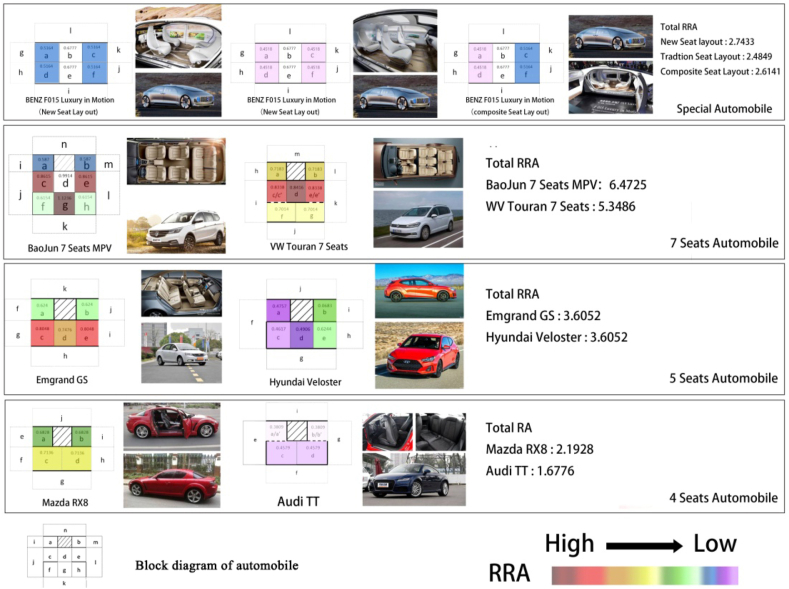
Comparison between the results of perception experiments and computed values of spatial syntax.

As previously mentioned, the RRA value calculated by the space syntax is the characterization of the fluidity of the interior space of the automobile. Based on Table 2, the vehicle fluidity data obtained by the expert group in the perception experiment for the space layout of an automobile interior were selected and compared with the RRA values of the corresponding vehicles in Table 4 (Fig. 6). As shown in Fig. 6, for the fluidity of automobiles with the same number of seats but different layouts, the evolution trend of the fluidity obtained by both methods was completely compatible.

### 3.2 Automobile seat layout analysis

The total standardized integration degree indicates the degree of tightness between nodes and all nodes in the entire system [19]; the higher the integration value, the lower the degree of tightness. The higher the depth value, the longer the topological distance from a point in space to the central space, the worse the spatial fluidity, and the better the privacy is. On the other hand, the total standardized integration degree is the reciprocal of the whole integration degree; the greater the standardized integration degree value, the better the liquidity.

Based on the driver's seat as the starting point in the interior space, the depth and standardized integration values of the seven automobile models are listed in Table 4.

**Fig. 7:**
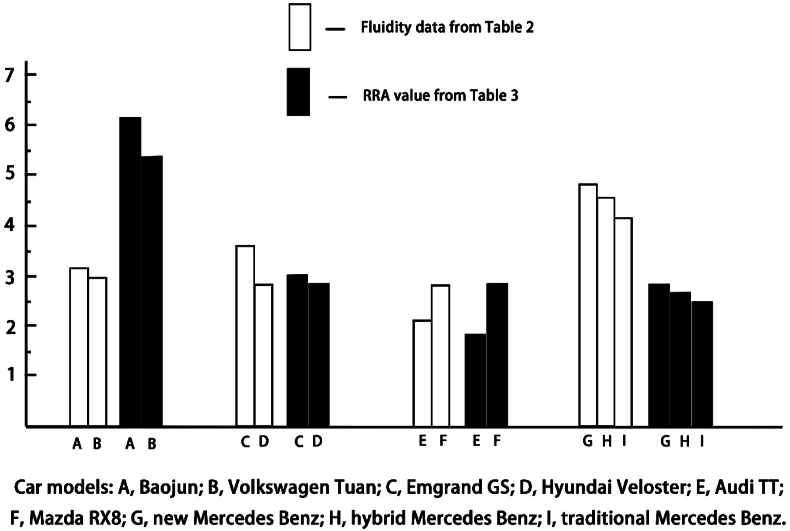
Distribution diagram of standardized integration degree of each automobile.

The results of the analysis of liquidity of the seven types of automobiles are reported in Table 4. The standardized integration values (indicated by the depth of the color) provide a certain intuition. Accordingly, we created the thermodynamic diagram of standardized integration values for the different automobile interiors (Fig. 7), which was helpful for the vertical comparison and evaluation of the standardized integration degree in the interior space of a single model and the horizontal comparison and evaluation of each interior unit in multiple models.

By combining Table 4 and Fig. 7, the following information can be obtained:

(1) For 7-seat Baojun 730 and 7-seat Volkswagen Touran, the total standardized integration values are 6.2455 and 5.3486, respectively. The standardized integration value of the Baojun 730 was greater than that of the Volkswagen Touran, and the permeability and liquidity of the interior space were better than that of the 7-seat Volkswagen Touran, in which it was more convenient for passengers to communicate with each other and reach a certain position in the automobile than in the Volkswagen Touran. However, the privacy of the interior space of the Baojun 730 was weaker than that of the Volkswagen Touran. From Fig. 7, for points close to the center of the interior space, their colors were the deepest, thereby indicating that their *RRA* values were higher. Therefore, in the case of keeping the number of seats in the automobile interior, setting the seats around the center of the interior space as far as possible would help improve the fluidity of the space and facilitate information exchange between passengers.
(2) For the 5-seat automobiles, the total standardized integration degrees of Emgrand GS and Hyundai Veloster space were 3.0652 and 2.7354, respectively. Hyundai Veloster has one fewer door than Emgrand GS and its spatial fluidity is obviously weaker than that of Emgrand GS. The same situation also occurred in the case of Audi TT (total RRA: 1.6776) and Mazda RX8 (total RRA: 2.7297) with 4-seats. The liquidity of Mazda RX8 was obviously better than Audi TT because it is a 4-door automobile, while Audi TT had only two doors. Clearly, the shape of the automobile actually has a profound impact on the “accessibility” (liquidity) of the automobile interior. As previously mentioned, the exterior of an automobile is an extension of the interior, and the shape of the exterior affects the spatial perception of the interior space.
(3) For the traditional, hybrid, and new layouts of the Mercedes-Benz driverless concept automobile, the total *RRA* values were 2.4849, 2.6141, and 2.7433, respectively. According to traditional automobile modes, interior spaces with better liquidity have worse privacy. Interestingly, the new Mercedes Benz F015 Luxury in Motion model has the largest RRA value and the best liquidity among the three concept automobiles. This may be because when the layout of the seats is the same, the change in the relative position among the seats leads to a difference between liquidity and privacy. Although the seats are arranged in the same way, the seats of the new Mercedes Benz can rotate freely. Therefore, the fluidity and privacy of the automobile interior space are related to the orientation of the seats, which is reflected in their relative orientation to facilitate communication between passengers.

Audi TT, Mazda RX8, and Mercedes Benz driverless concept automobile are all 4-seat automobiles and the order of their total *RRA* value is new Mercedes Benz > Mazda RX8 > hybrid Mercedes Benz > traditional Mercedes Benz > Audi TT. The total *RRA* values of the new Mercedes Benz and Mazda RX8 models were almost the same at 2.7433 and 2.7297, respectively. The total *RRA* of the Mercedes Benz F015 Luxury in Motion with the hybrid and traditional layouts was slightly smaller than that of the Mazda RX8. This may be because the Mazda RX8 has four doors, which is convenient for getting into the vehicle from different positions.

Based on the analysis of the above data combined with the perception experiment of the automobile interior space, the following conclusions were drawn.

A) The results of the fluidity and privacy of the interior space of these seven models of automobile obtained via the space syntax method were basically consistent with the results of the perception experiment of the layout of automobile interiors, which indicated that the space syntax was feasible as an auxiliary tool for the space design of automobile interiors and provided designers with credible numerical reference.
B) To facilitate passengers' activities in the automobile, a variable space could be considered in the interior design of the automobile; for example, design of the rotatable seat was an effective method to achieve the goal of good fluidity of the automobile space.
C) The better the fluidity of the space, the more convenient communication among the passengers, and the better the openness of the space. By contrast, better openness means less or reduced privacy.
D) For automobiles with the same number of seats, communication between the interior space and the surrounding environment should be facilitated by increasing the communication channel between the interior and exterior space, for example, by setting appropriate automobile doors.

## 4 Conclusions

Based on a questionnaire survey method, this study conducted a perception experiment of automobile interior space to investigate the effect of layout of seats on the fluidity and privacy of interior space. The perception difference between ordinary users and designers was investigated and the results revealed little difference between these two groups for the fluidity and privacy of the automobile interior space owing to the different backgrounds of the participants.

The space syntax method was introduced into the study of seat layout design. A total of seven models of automobile interior with different seats were taken as examples to obtain corresponding depth and standardized integration values. The results indicates the following: (1) In the process of automobile interior design, setting the seats as rotatable and other forms can improve the fluidity of automobile interior space. (2) The greater the total standardized integration value of automobiles, the more compact the space structure of the automobile interior, and the better the space permeability. (3) For the design of automobile interiors with the same number of seats, fewer doors means that the communication between the interior space and the surrounding environment space was inconvenient and was not conducive to the fluidity of the interior space. (4) The calculation results of spatial syntax analysis are consistent with the experimental results of perception of automobile interior space layout regarding the fluidity and privacy. The calculation results of spatial syntax were basically consistent with the experimental results of automobile interior spatial perception, indicating that the calculation results were consistent with the user's cognitive results, thereby proving that spatial syntax is a feasible auxiliary tool for automobile interior spatial design. Spatial syntax is an effective design analysis tool for fluidity and privacy of automobile interior space.

In this study, we only adopted spatial syntax to examine the impact of seat layout on the fluidity and privacy perception of automobile interior space. According to the perception experiment and the calculation of the automobile interior space based on space syntax, it can be expected that, in design practice, various variables can be configured according to different design requirements, different topological unit sizes can be selected, and spatial syntax can be used to assist in design. Although the applicability of spatial syntax is not explicitly constrained by the scale and numerical values of spatial dimensions, the fundamental concept of spatial syntax lies in describing human movement in space and the relationships among various elements within space through topological relations. This means that spatial syntax is not applicable to the research and design of interior spaces related to manned spaces with lacking dynamic feature and too small topological depth [14,24] (such as single tower crane operation rooms). However, in the future, the space syntax tool can provide more options for design practice by matching, modifying, and iterating industrial designs, and become a significant step in the automobile interior design process. Specifically, we anticipate that spatial syntax will play an important role in the interior layout design of industrial products with larger interior spaces, such as large aircraft and transport vehicles.

## Data availability statement

Data included in article/referenced in article.

## Declaration

This article does not involve any animal experiments or ethical issues. Not involving the interests and personal privacy of the participants in the experiments.

## CRediT authorship contribution statement

**Yingjie Huang:** Writing – original draft, Resources, Data curation, Conceptualization. **Danhua Zhao:** Visualization.

## Declaration of competing interest

The authors declare the following financial interests/personal relationships which may be considered as potential competing interests: Yingjie Huang reports was provided by Ministry of Science and Technology of the People's Republic of China and Science and Technology Commission of Shanghai Municipality. If there are other authors, they declare that they have no known competing financial interests or personal relationships that could have appeared to influence the work reported in this paper.
